# Racial Assumptions Color the Mental Representation of Social Class

**DOI:** 10.3389/fpsyg.2017.00519

**Published:** 2017-04-05

**Authors:** Ryan F. Lei, Galen V. Bodenhausen

**Affiliations:** ^1^Department of Psychology, Northwestern University, EvanstonIL, USA; ^2^Department of Marketing, Kellogg School of Management, Northwestern University, EvanstonIL, USA

**Keywords:** social class, race, face perception, social cognition, economic attitudes, dehumanization

## Abstract

We investigated the racial content of perceivers’ mental images of different socioeconomic categories. We selected participants who were either high or low in prejudice toward the poor. These participants saw 400 pairs of visually noisy face images. Depending on condition, participants chose the face that looked like a poor person, a middle income person, or a rich person. We averaged the faces selected to create composite images of each social class. A second group of participants rated the stereotypical Blackness of these images. They also rated the face images on a variety of psychological traits. Participants high in economic prejudice produced strongly class-differentiated mental images. They imagined the poor to be Blacker than middle income and wealthy people. They also imagined them to have less positive psychological characteristics. Participants low in economic prejudice also possessed images of the wealthy that were relatively White, but they represented poor and middle class people in a less racially differentiated way. We discuss implications for understanding the intersections of race and class in social perception.

## Introduction

Recent psychological research on social categorization has emphasized the intersecting and overlapping nature of our representations of social groups (Cole, 2009; Ridgeway and Kricheli-Katz, 2013; Kang and Bodenhausen, 2015). From this perspective, the meaning attached to particular category memberships can vary as a function of other social identities (e.g., gender stereotypes may differ as a function of perceived race; Shields, 2008), and membership in a particular social category may lead to the presumption of membership in other categories that are assumed to covary with it (e.g., members of high-status occupational groups are often assumed to be men; Glick et al., 1995). In the present research, we examine the connections between mental representations of race and social class. Specifically, we examine the hypothesis that conceptions of social class are racialized, with low economic status being associated with Black Americans and high economic status being associated with White Americans.

As Sanchez and Garcia (2012) have noted, surprisingly little research has directly examined the connections between race and class in the minds of social perceivers. Given that White individuals constitute the majority group in the United States and thus make up the greatest percentage of both poor and rich individuals (DeNavas-Walt and Proctor, 2015), one reasonable possibility is that the racial content of all social class categories, including the poor, is White by default. This view accords with the assertion of intersectionality theorists that when perceivers think about one subordinated social category (e.g., the poor), they nevertheless assume default features on other dimensions (e.g., White, male, heterosexual, etc.; Purdie-Vaughns and Eibach, 2008). However, other research suggests that there may be a psychological association between subordinated race and class identities. Specifically, using an explicit group description task, Cox and Devine (2015) found evidence that when people think of the category “Black person” they tend to activate the concept of being poor; however, they did not find evidence for the converse pattern. That is, when people think of the category “poor person” they do not generally activate the concept “Black.” Overall, this suggests that mental representations of the poor may not incorporate any inherent notion of Blackness.

Nevertheless, it seems premature to reject the possibility that class is indeed racialized, given other research suggesting connections between race and social class. For example, Fiske et al. (2002) utilized judgments on the dimensions of competence and warmth to characterize the fundamental content of the stereotypes of different groups. Their results showed that when people consider both ingroups and outgroups, the category “Middle Class People” was located very close to the category “Whites” in this two-dimensional space, while the categories of “Blue-Collar People” and “Blacks” were close neighbors. Thus, the core content of social class stereotypes overlaps substantially with that of race stereotypes. In a quite different paradigm, Penner and Saperstein (2008; Saperstein and Penner, 2012) showed that when people experience changes in their socioeconomic status, it changes their likelihood of being categorized as Black or White by others and even by themselves. Specifically, factors such as becoming unemployed resulted in an increased likelihood of being categorized as Black, and this was true both for women and men (Penner and Saperstein, 2013).

Most relevant to the hypothesis that social class is racialized is a recent set of experiments by Brown-Iannuzzi et al. (2017). These studies showed that perceivers’ mental representations of welfare recipients were significantly Blacker than their representations of non-welfare recipients. This finding comports with the possibility that social class is indeed racialized in the minds of social perceivers, but it also raises important questions that require further exploration. First, “welfare recipients” can be regarded as a subset of the broader category of “poor people,” and it is not clear how interchangeable the two concepts are. In particular, the term “welfare recipients” has come to serve as a “racial dog-whistle” in American politics; that is, it is a common strategy whereby communicators invoke notions of race without explicitly mentioning it (e.g., Gilens, 1996; Haney-López, 2015). It is thus unclear whether a more neutral term for the poor would evoke the same racialized mental representations. Second, “non-welfare-recipients” is a large and heterogeneous comparison category, and it would be of interest to specifically examine whether there are racial distinctions made between representations of middle class and upper-class groups. In addition to addressing these open questions, it seems likely that the tendency to racialize mental representations of the poor is not uniform; rather, it likely varies as a function of social attitudes. In the present research, we explored the possibility that people who hold the poor in greater contempt are also likely to mentally represent the poor as stereotypically Blacker than more economically advantaged social classes.

We employed the method of reverse correlation (e.g., Todorov et al., 2011) to examine the question of whether thinking about different social classes involves assumptions about race. The reverse correlation technique consists of two phases: an image-generation phase and an image-rating phase, described in more detail in the Section “Materials and Methods”. Past research confirms that the reverse correlation procedure captures psychologically meaningful patterns of mental representation and can be sensitive to individual difference factors. For example, Dotsch et al. (2008) found that representations of outgroup faces (Moroccans for Dutch participants) were more criminal-looking and less trustworthy for prejudiced participants, versus less prejudiced participants. For this reason, we measured our participants’ attitudes toward the poor (Stevenson and Medler, 1995) several weeks prior to their completion of the image-generation phase of the reverse correlation task. Notably, the items in the questionnaire make no mention of race whatsoever. We examined whether variations in the amount of contempt expressed toward the poor on this explicit measure would be related to the tendency to imbue social class with racial content.

## Materials and Methods

We report all measures, manipulations, and data exclusions. The method involved two phases, one in which mental images of different social classes were assessed, and one in which a separate group of participants evaluated these images. Overall, the study consists of a 3 (target social class: poor, middle class, and rich) × 2 (economic prejudice of the image generators: low vs. high) design. For the image generation phase, this is a between-subjects design. For the image rating phase, this becomes a fully within-subjects design.

### Image Generation Phase

#### Participants

Prior experiments on visual representation of outgroup faces by Dotsch et al. (2008) and Dotsch and Todorov (2012) used between 15 and 35 participants for the image generation phase; they repeatedly documented individual differences in visual representations within these samples. We thus aimed to collect 35 participants per each of our three social class conditions, resulting in a final sample of 107 participants. Participants were students in an introductory psychology class who received course credit. They were on average 18.65 years old (*SD* = 1.25) and 41% male. Most participants self-identified as White (41%) or Asian (38%), with the rest identifying as African American (8%), Latino (8%) or multiracial (4%). Their average subjective self-rating of socioeconomic status (Adler et al., 2000) was 6.79 (*SD* = 1.82) on a 10-point scale, with higher scores indicating higher subjective socioeconomic status (SES).

#### Procedure

Participants completed questionnaire items concerning their explicit attitudes toward the economically disadvantaged (Stevenson and Medler, 1995) at a mass testing session 4–8 weeks prior to their participation in the laboratory. This 9-item Likert scale assesses agreement with negative statements about poor people (e.g., “People who do not make much money are generally unmotivated.”) and is scored such that higher scores indicate more negativity toward the poor (α = 0.94). The response scale ranges from 1 (strongly disagree) to 5 (strongly agree). Individuals were selected to participate in the study based on their scores on this measure; based on the overall distribution of scores, we chose participants who scored in the upper third of responses (*M* = 3.05, *SD* = 0.50) for the high-prejudice group and those who scored in the lower third of the distribution (*M* = 1.29, *SD* = 0.28) for the low-prejudice group.

Participants reported to a laboratory for the main experimental session. They were randomly assigned to one of three conditions – to choose either the “poor,” “middle income,” or “rich” person during the image generation task. Prior to the lab sessions, a biracial base photo (Krosch and Amodio, 2014) was superimposed with sinusoidal noise patterns and the inverse of the noise patterns (Dotsch and Todorov, 2012) repeatedly in order to generate 400 pairs of face-images that were presented to participants one pair at a time on a computer screen in a private room. On each trial, they were simply asked to choose which of the two pictures best matched the relevant criterion (e.g., “which face looks poor?” in the poor condition); notably, race was never mentioned during this image-generation phase. Averaging all of the participant’s selections resulted in a composite image of what the participant thought a poor, middle income, or rich person looked like, depending on condition. The composite images from each participant within a condition were then averaged to generate an overall composite image for that condition, separately for the participants low versus high in economic prejudice, resulting in a total of six final composite images.

### Image Rating Phase

#### Participants

During the image rating phase, a new set of participants recruited from Amazon’s Mechanical Turk were asked to rate all of the composite images generated by the lab participants. These participants were naïve to the conditions under which the composite images were created. They received $1 in return for completing a brief online survey. We sought a sample of *N* = 90 to provide adequate power (1-β > 0.80) to detect a medium-sized effect (*r* = 0.30; G^∗^Power software, Faul et al., 2009). Three participants were excluded due to excessive missing data, one did not provide affirmative consent, and two indicated that they were not careful while taking the survey in a quality check included at the very end of the study. The final sample consisted of 84 participants. The sample was 54% female, mostly White (71%), had a mean age of 32.71 (*SD* = 10.53), and a mean subjective self-rating of SES of 5.26 (*SD* = 1.78).

#### Procedure

Participants were told that the researchers were interested in seeing how well they could discern information from blurry photos. They were asked to rate the six images created during the image-generation phase on 15 different trait dimensions. The order of image presentation was randomized. The primary dependent variable was stereotypical Blackness. We also included skin tone as another measure of racial categorization. Additionally, based on work examining the stereotype content of poor individuals, we also assessed stereotypes such laziness, motivation, and intelligence (Fiske et al., 2002), as well as a question about how well-groomed participants thought the depicted person looks. Next, based on work suggesting that low-SES individuals are dehumanized, we also assessed a group of traits that have been considered to reflect the degree of perceived humanity of social targets (e.g., curiosity and refinement; Haslam, 2006; Loughnan et al., 2014). Participants saw all six images in a randomized order and were asked to provide their ratings (1 = not at all, 6 = completely) on each of the trait dimensions. Finally, participants were shown each of the six images again in a new random order, and they were asked to categorize the race of each image. Specifically, they were given a multiple-choice question that asked, “Does the person in this picture look mostly: [Black, Asian, White, Latino, or Native American]?” After completing these ratings, participants provided their own demographic details and were then debriefed and paid.

## Results

**FIGURE 1:**
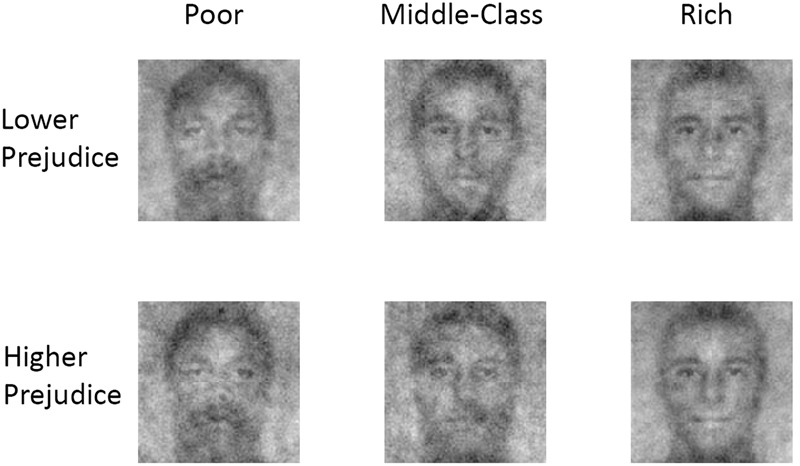
**Composite images generated by participants.** The top row shows the composite images selected by the relatively more economically prejudiced participants as representing poor (1a), middle income (1b), or rich (1c) faces. The bottom row shows the composite images of relatively less prejudiced participants for the corresponding social classes.

Composite images for each of the six combinations of class condition × economic prejudice level of the image generators are shown in **Figure 1**.

A repeated-measures ANOVA examining ratings of stereotypical Blackness as a function of participants’ prejudice level (low or high) and condition (poor, middle income, or rich) revealed a significant main effect of SES category, *F*(2, 164) = 22.20, *p* < 0.001, $\eta_{p}^{2}$ = 0.21, such that the poor composite (*M* = 2.97, *SD* = 1.56) was rated as significantly more stereotypically Black than either the middle income (*M* = 2.20, *SD* = 1.10) or rich (*M* = 2.06, *SD* = 1.24) composite, both *p*s < 0.001, with no difference between the latter two composites, *p* = 0.55. Results also revealed a significant main effect of the economic prejudice level of the participants generating the image, *F*(1,82) = 4.63, *p* = 0.009, $\eta_{p}^{2}$ = 0.08, such that the composites generated by the relatively more prejudiced participants (*M* = 2.31, *SD* = 1.06) were slightly less stereotypically Black than the composites generated by participants who were less prejudiced (*M* = 2.51, *SD* = 1.15; *p* = 0.009).

Notably, there was also a significant interaction between SES category and level of economic prejudice of the participants generating the composite image, *F*(2,164) = 12.21, *p* < 0.001, $\eta_{p}^{2}$ = 0.13. Among the composites generated by relatively more prejudiced participants, the poor composite (*M* = 3.11, *SD* = 1.71) was rated as significantly more stereotypically Black than either the middle income composite (*M* = 1.86, *SD* = 1.17; *p* < 0.001) or the rich composite (*M* = 1.98, *SD* = 1.30; *p* < 0.001). Among the composites generated by the relatively less prejudiced participants, there was no significant difference in the stereotypic Blackness ratings for the poor (*M* = 2.83, *SD* = 1.68) versus middle income (*M* = 2.54, *SD* = 1.37; *p* = 0.33) composites; however, both were rated as more stereotypically Black than the rich composite (*M* = 2.15, *SD* = 1.38), *p*s = 0.002 and 0.025, respectively. Examined differently, high-prejudice participants generated a composite image of the poor that was directionally higher in Blackness than did their low-prejudice counterparts, but the difference did not reach statistical significance, *M_diff_* = 0.277, *p* = 0.057. There was no difference in the rated Blackness of the images of the rich as a function of economic prejudice, *M_diff_* = –0.169, *p* = 0.145. However, for the middle income composite, low-prejudice participants generated an image that was rated as more stereotypically Black than the one generated by more prejudiced participants (*M_diff_* = 0.687, *p* < 0.001).

**FIGURE 2:**
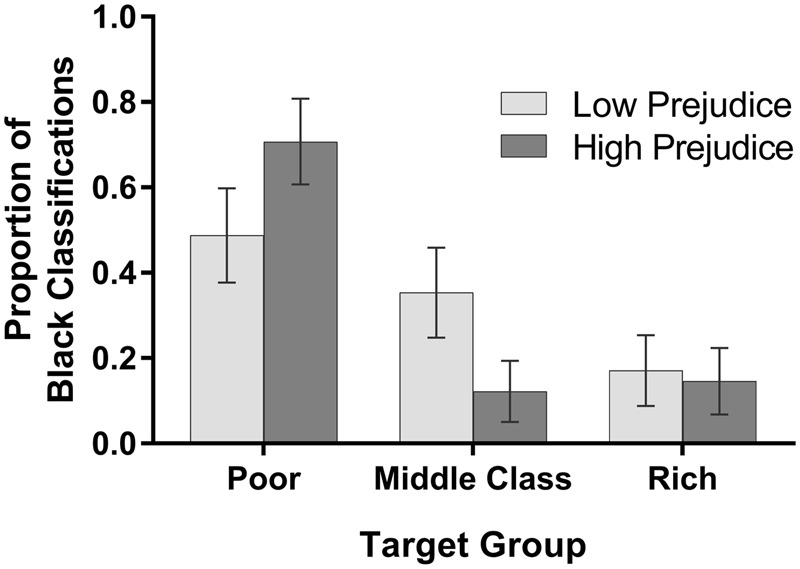
**Black classifications of composite images.** Mean proportions (and 95% confidence intervals) of categorizing the facial image as Black.

For the racial categorization task, we first created a dichotomous variable reflecting whether or not the image raters classified a given composite image as Black. The proportions of “Black” classifications are shown in **Figure 2** as a function of the target’s social class and the economic prejudice level of the image generators. Results paralleled the stereotypic Blackness trait ratings. We analyzed this index using a logit mixed-model approach, given the dichotomous nature of this variable (e.g., Jaeger, 2008). This analysis confirmed a significant effect of target group, *F*(2,496) = 35.51, *p* < 0.001, such that poor images were most likely to be categorized as Black, rich images were least likely to be categorized as Black, and middle class images fell in between. This main effect was qualified by a significant interaction with the prejudice level of the image generators, *F*(2,496) = 18.02, *p* < 0.001. Decomposing this interaction, we see that although the rich images were equally unlikely to be seen as Black regardless of prejudice level, *p* = 0.81, prejudice level *did* influence the likelihood that poor and middle class images looked Black. Specifically, the poor images of high-prejudice people were significantly more likely to be categorized as Black than those of low-prejudice people, and the middle class images of high-prejudice people were significantly less likely to be categorized as Black than those of low-prejudice people, *p*s < 0.001. Examined differently, the high-prejudice image generators produced substantially more Black-looking images of the poor than of the middle class or rich, *p*s < 0.001, but their images of the middle class and rich were equally un-Black-looking, *p* = 0.49. Low-prejudice image generators, in contrast, produced images of the poor that were only somewhat more Black-looking than their images of the middle class (*p* = 0.045), but their mental image of the middle class was substantially more Black-looking than their image of the rich (*p* < 0.001). In other words, low-prejudice individuals imputed relatively similar levels of Blackness to the poor and middle class, whereas high-prejudice individuals imputed much more Blackness to the poor than to the middle class.

**FIGURE 3:**
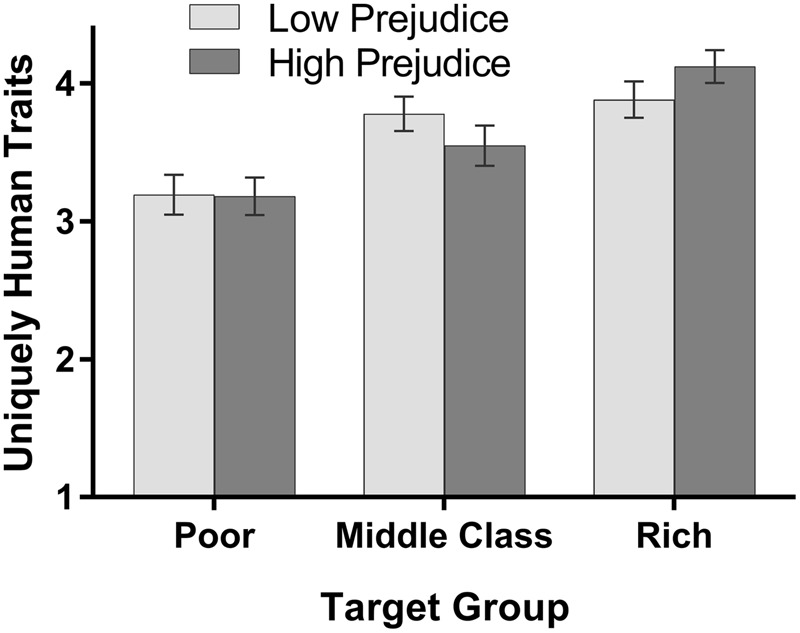
**Perceived humanity of composite images.** Mean ratings (and 95% confidence intervals) of the humanness of the representations of the social classes as a function of economic prejudice.

To assess the extent of dehumanization in mental representations of social class, we collected ratings of 8 traits that are associated with “humanness” (see Haslam, 2006; Bastian and Haslam, 2010): unrefined (R), rational, cultured, polite, disorganized (R), impulsive (R), irresponsible (R), and curious, with higher scores reflecting greater perceived humanness. The scale exhibited acceptable reliability (average α = 0.69). Results are depicted in **Figure 3**. As the figure indicates, there was a main effect of social class condition, *F*(2,133.42)^1^ = 60.04, *p* < 0.001, $\eta_{p}^{2}$ = 0.42, which was further qualified by a significant interaction of social class condition and economic prejudice, *F*(2,151.89) = 9.93, *p* < 0.001, $\eta_{p}^{2}$ = 0.11. Images generated by participants with higher levels of economic prejudice were seen by naïve raters as possessing greater humanity as SES increased; they represented middle income people as having greater humanity than poor people, and rich people as having greater humanity than middle income people (*p*s < 0.001). Images of the middle class generated by participants with lower levels of economic prejudice were also seen by naïve raters as possessing greater humanity than poor people (*p* < 0.001), but there was no difference in the humanness of the middle class and rich images (*p* = 0.56). Examined differently, poor people were represented as similar in their humanity by those high vs. low in economic prejudice (*p* = 0.83), but high-prejudiced individuals represented middle income people as less human (*p* = 0.008) and rich people as more human (*p* = 0.001) than did low-prejudice individuals.

Remaining results are summarized in **Table 1**. In general, the ratings indicate that all individuals represent the social classes in psychologically differentiated ways, although the differentiation is generally stronger among those who are higher in economic prejudice. Overall, participants visualized poor people as less friendly, less intelligent, lazier, less motivated, and less well-groomed than wealthier people.

**Table 1:** Means for ratings of composite images as a function of targeted social class and economic prejudice level; inferential statistics for the social class × economic prejudice interaction are presented in the right columns.

Measure	Lower economic prejudice			Higher economic prejudice			Interaction	
	Poor	middle income	Rich	Poor	middle income	Rich	*F*	*p*
Dark-Skinned	3.57^a^	3.30^a^	2.75^b^	3.69^a^	2.57^b^	2.62^b^	6.81	0.001
Friendly	2.31^a^	3.55^b^	4.01^c^	2.38^a^	3.33^b^	4.62^c^	8.70	0.001
Intelligent	2.86^a^	3.31^b^	3.65^c^	2.76^a^	3.23^b^	4.03^c^	4.97	0.008
Lazy	3.33^a^	2.47^b^	2.47^b^	3.14^a^	2.78^ab^	2.41^b^	3.46	0.034
Motivated	2.52^a^	3.26^b^	3.64^c^	2.61^a^	3.08^b^	3.98^c^	4.52	0.012
Weil-groomed	2.24^a^	3.80^b^	4.11^c^	1.95^a^	3.03^b^	4.68^c^	25.62	0.001

*Within prejudice level, means with different superscripts are significantly different from one another, *p* < 0.01.*

## Discussion

These results support the hypothesis that social class is racialized. Among relatively more class-prejudiced participants, the representation of a poor person was Blacker than the representations generated for the middle income or rich composites. While the poor composite was infused with greater Blackness relative to the middle income and rich composites, the composites representing the latter two classes were rated as similarly White. In contrast, images of the poor and middle class generated by less class-prejudiced participants were relatively less racially differentiated, although like their high-prejudice counterparts, they also represented the wealthy in White-looking ways.

These findings expand on the earlier research of Brown-Iannuzzi et al. (2017) in some noteworthy ways. They show that racialized representations of the poor can be found even in the absence of racial dog-whistle terms such as “welfare recipients,” they confirm that wealthy people are imagined to look White, and they show that the racial content of mental images of the poor and the middle class vary depending on level of economic prejudice. In this latter regard, the findings confirm that economic prejudice can moderate the racialization of social class.

These facial representations also carried noteworthy psychological connotations. The poor composite generated by the relatively more prejudiced participants was perceived as less likely to possess positive traits (e.g., uniquely human qualities) and more likely to possess negative traits (e.g., laziness). In addition, assessments of the humanity of the facial composites provided evidence that there are some respects in which the wealthy are represented in psychologically more favorable ways than the middle class. That these results emerged differentially for relatively more prejudiced versus relatively less prejudiced participants is also consistent with work by other researchers that find attitudes can influence the representations of category prototypes or exemplars that come to mind (Dotsch et al., 2008; Young et al., 2014).

This study indicates that social class connotes race, such that the category “poor people” is mentally represented as relatively Black, even though there are many more poor White than poor Black people in the US. People also imagine the poor to be lower in distinctly human traits, less friendly, less intelligent, and in general as less psychologically fit than middle income or rich groups. The tendency we observed for people to imbue social class representations with racial content stands in contrast to the recent findings of Cox and Devine (2015), who found that people do not spontaneously think of Black as being associated with the concept “poor person.” Of course, there is a major difference in the methodology involved in the two studies. The research of Cox and Devine examined participants’ explicit statements about the characteristics of “poor people,” whereas our method involved no explicit statements at all. It may be that people are reluctant to explicitly state that poor people are Black, or they may not explicitly realize the extent to which blackness is a feature of their mental representation of the poor.

Our results comport with the notion that resentments regarding policies designed to help the poor are tied up with racial prejudice (e.g., Gilens, 1996). People who view the poor as lazy and parasitical tend to mentally represent the category “poor” as blacker and the category “rich” as whiter, compared to the people who are low in contempt for the poor. In addition to the racism that underlies the disinclination to help the (presumptively Black) poor, Ridgeway and Kricheli-Katz (2013) argued that poor Whites are also put in a bind by virtue of not being prototypically of the poor category. McDermott (2010) reports fascinating evidence of poor Appalachian Whites who choose to identify as “Black” (based on uncertain, distant minority ancestry) in order to avoid the shame of being poor Whites.

One major limitation of the current work is that we had a primarily White and Asian sample. Both of these racial groups are higher status groups in society (cf. Fiske et al., 2002), and thus may share similar mental representations of SES categories. In contrast, lower-status groups may have different representations of SES, because they are trying to increase their group’s position in the social hierarchy. Further research is needed to better understand this potential divergence among high versus low status individuals.

Overall, our results confirm that class is racialized. Those who express more negative attitudes toward the poor have clearer racial delineations depending on SES category, compared to those who express more generous attitudes toward the poor. One interesting direction for future research will be to examine the effects of situational manipulations relating to the perceived causes of poverty and the nonracial characteristics of poor people on the racial content of mental representations of social class.

## Ethics Statement

This study was carried out in accordance with the recommendations of the Institutional Review Board (IRB) at Northwestern University with written informed consent from all subjects. All subjects gave written informed consent in accordance with the Declaration of Helsinki. The protocol was approved by the Northwestern IRB.

## Author Contributions

Conceived and designed the experiment: RL and GB. Performed the experiment: RL. Analyzed the data: RL and GB. Wrote the paper: RL and GB.

## Conflict of Interest Statement

The authors declare that the research was conducted in the absence of any commercial or financial relationships that could be construed as a potential conflict of interest.

## Footnotes

1 Mauchly’s test of sphericity was significant (indicating violation of sphericity), so we used the Greenhouse–Geisser adjustment for all analyses.
